# Diffuse multiple scattering

**DOI:** 10.1107/S2053273314026515

**Published:** 2015-01-01

**Authors:** A. G. A. Nisbet, G. Beutier, F. Fabrizi, B. Moser, S. P. Collins

**Affiliations:** a Diamond Light Source, Harwell Science and Innovation Campus, OX11 0DE, UK; b CNRS, SIMAP, F-38000 Grenoble, France; c Univ. Grenoble Alpes, SIMAP, F-38000 Grenoble, France

**Keywords:** diffuse scattering, multiple scattering, Kossel lines, Kikuchi lines, strain, lattice parameters

## Abstract

A new form of diffraction lines has been identified, similar to Rutherford, Kikuchi and Kossel lines. This paper highlights some of the properties of these lines and shows how they can be used to eliminate the need for sample/source matching in Lonsdale’s triple convergent line method in lattice-parameter determination.

## Abstract 2

A new form of diffraction lines similar to Rutherford, Kikuchi and Kossel lines has been identified. They can be used to eliminate the need for sample/source matching in Lonsdale’s triple convergent line method in lattice-parameter determination.

## 1. Introduction   

We present a new technique for structural analysis in single crystals, extending the capabilities of existing multiple-scattering methods. Lines similar to Rutherford, Kikuchi and Kossel lines arising from incoherent X-ray multiple-scattering processes have been observed for numerous materials. We will present data from Cu and Au samples.

In multiple-wave diffraction, the interaction between diffracted waves can lead to an increase or decrease in the measured intensity. When the intensity is decreased due to the energy being diverted to other reflections, the intensity reduction is called *aufhellung* and was first reported by Wagner (1920 ▶) and then by Berg (1926 ▶). On the other hand, when a secondary reflection acts as the source for a tertiary reflection emerging in the direction of the primary reflection, the intensity can be modified by constructive or destructive interference. This is known as *umweganregung* (*umweg* for short) and was first reported by Renninger (1937 ▶). If the incident beam is collimated, the multiple diffraction emerges as diffraction spots. If, however, the source is an external divergent source close to the sample, the observed diffraction produces lines. This effect was first demonstrated as far back as 1914 by Rutherford & Andrade (1914 ▶), closely followed by Seemann (1916 ▶) and later by Fujiwara (Fujiwara, 1928 ▶; Fujiwara & Onoyama, 1937 ▶, 1939 ▶) and Lonsdale (1947 ▶). These lines were first referred to as pseudo-Kossel lines by Imura (1954 ▶), which was somewhat erroneous considering they pre-dated Kossel’s technique. The term, however, has persisted (Tixier & Waché, 1970 ▶; Okada & Iwasaki, 1980 ▶; Lang, 1995 ▶; Chang, 2004 ▶; Cowley, 1975 ▶). The same effect can be achieved using an internal source, as demonstrated by Kossel (1935 ▶). Kossel reported a method in which he and his colleagues used a single crystal as an anti-cathode in an X-ray tube to excite characteristic divergent X-rays within the crystal. (Kossel, 1935 ▶; Chang, 2004 ▶; Novikov *et al.*, 1998 ▶; Cowley, 1975 ▶; Lonsdale, 1947 ▶). Borrmann used a similar method but placed the crystal outside the tube, using the higher-energy X-rays from the tube to excite the lower-energy fluorescence in the crystal (Borrmann, 1935 ▶, 1936 ▶). Kikuchi observed similar lines with an electron beam, which he attributed to the divergence of the cathode rays, reporting the similarity with the work of Rutherford and Andrade using γ-rays (Kikuchi, 1928 ▶).

We aim to extend the story of diffraction lines by proposing a fourth process which has become apparent a century on from Rutherford’s experiment only because of the immense flux of third-generation synchrotron sources and the considerable dynamic range of modern area detectors. While sharing characteristics of Kikuchi, Kossel and pseudo-Kossel lines, the source of the lines is different. The incident beam is not divergent and neither is it tuned to the characteristic energy of the elements within the crystal. Instead, the divergent source is provided by the diffuse scatter arising from a disruption in the long-range order of the crystal such as structural defects or crystal-surface truncation. As the lines are distinct from Kossel lines, and in certain circumstances can be seen at the same time, we have introduced, for the sake of clarity, a new term, namely diffuse multiple scattering (DMS). DMS lines exhibit several exploitable differences from the above processes which will be explored in the current paper. In particular, we will extend the capabilities of Lonsdale’s method in the context of tunable synchrotron radiation. Finally, we will provide a convenient algorithm for calculating the geometry of the DMS features.

## 2. Geometrical model   

### 2.1. Multiple scattering   

**Figure 1:**
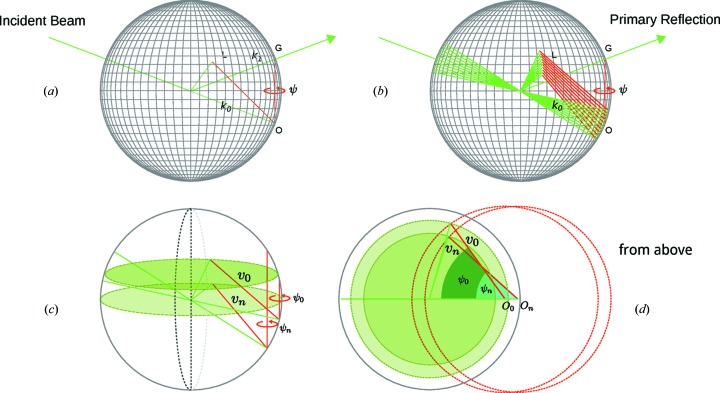
Part (*a*) shows the Ewald construction for the simple multiple-scattering case with scattering vectors *G* and *L* simultaneously intersecting the sphere [the reciprocal-lattice vectors are in red and the beam vectors are in green for (*a*) to (*d*)]. Part (*b*) shows the Ewald construction extended for diffuse multiple scattering. The reciprocal-space origin changes with the incident angle (we are now assuming multiple incident beams). Consequently, the positions at which the reciprocal-lattice vectors intersect the sphere are changed, producing lines. Parts (*c*) and (*d*) provide a simplified view of (*b*) at two different projections. 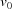 and 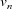 refer to the resulting vectors connecting the new origins to the surface of the sphere for a range of incident-beam vectors 0 to *n*.

In multiple scattering, the geometry at which multiple scattering occurs is determined by several parameters, namely, the energy of the incident beam, which fixes the size of the Ewald sphere, the direction of the primary scattering vector, the reciprocal-space origin, which is set by the primary Bragg angle, and an azimuthal reference with respect to which the azimuthal angles are defined (see Fig. 1 ▶ *a*). At its most basic, multiple scattering occurs when two or more reciprocal-lattice vectors simultaneously intersect the Ewald sphere. This will result in a secondary reflection emitted in a direction away from the primary scattered beam vector (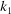). If the secondary and tertiary scattering vectors sum to the primary scattering vector (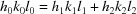), the beam will be redirected by the tertiary reflection in the direction of 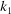 and effect an intensity change at the detector. A convenient method for measuring this is the Renninger scan (Renninger, 1937 ▶), in which the sample is rotated about a primary scattering vector. Finding the azimuthal angles at which the reciprocal-lattice vectors intersect the Ewald sphere can trivially be reduced to a problem of intersecting circles determined by the reciprocal-lattice vectors (*L*) and their corresponding Ewald slices. This is done by converting *hkl*’s to reciprocal-lattice vectors using the *B* matrix (Busing & Levy, 1967 ▶) and defining circles with their origins positioned along the primary reciprocal-lattice vector (*G*) and their axes parallel to *G*. The radius of the circles is given by the norm of the projection of *L* on the plane perpendicular to *G*. The radius of the Ewald slice is given by 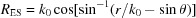, where *r* is the component of the reciprocal-lattice vectors projected onto *G* and θ is the Bragg angle of the primary reflection.

### 2.2. Diffuse multiple scattering   

DMS is almost identical to normal multiple scattering in terms of its geometry. If we extend the Ewald construction so we are no longer considering the integer case but have an extended source within the crystal arising from the diffuse scatter of a nearby Bragg peak or crystal truncation rod (CTR), we have the scheme depicted in Fig. 1 ▶(*b*). This is analogous to having a range of incident angles providing a continuous set of reciprocal-space origins. Calculating the azimuthal angles at which the crystal reciprocal-lattice vectors intercept the Ewald sphere for each of these origins results in lines across the sphere’s surface. Figs. 1 ▶(*c*) and 1 ▶(*d*) show 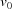 and 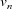 intersecting the sphere at different elevations and azimuths 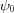 and 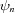, respectively.

**Figure 2:**
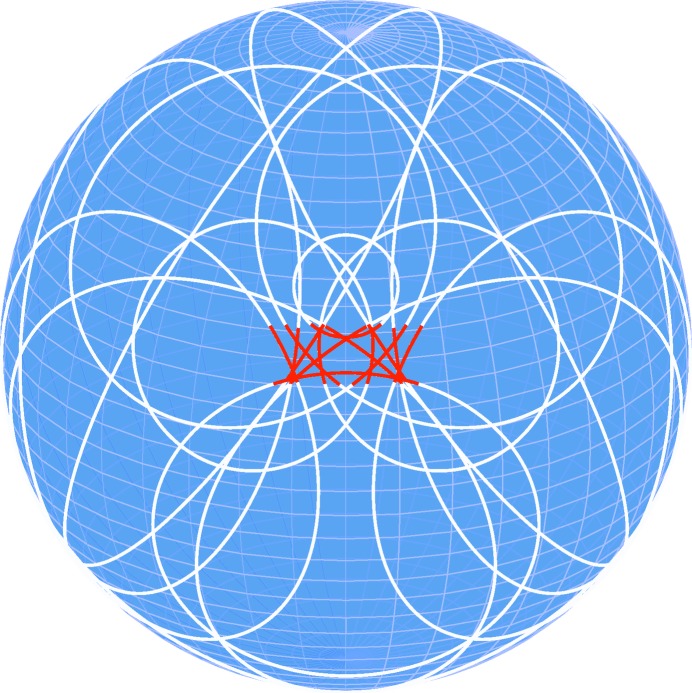
Comparison of calculated Kossel conics (white) with calculated DMS lines (red) for the Si 002 primary reflection at 6 keV, showing the geometrical equivalence of both methods.

Ignoring polarization, this is geometrically equivalent to defining an internal spherical wave which then produces conics where the Bragg condition is met. This equivalence is demonstrated in Fig. 2 ▶, which shows the calculated Kossel conics and DMS lines calculated according to the method described above for the same energy.

### 2.3. Polarization   

**Figure 3:**
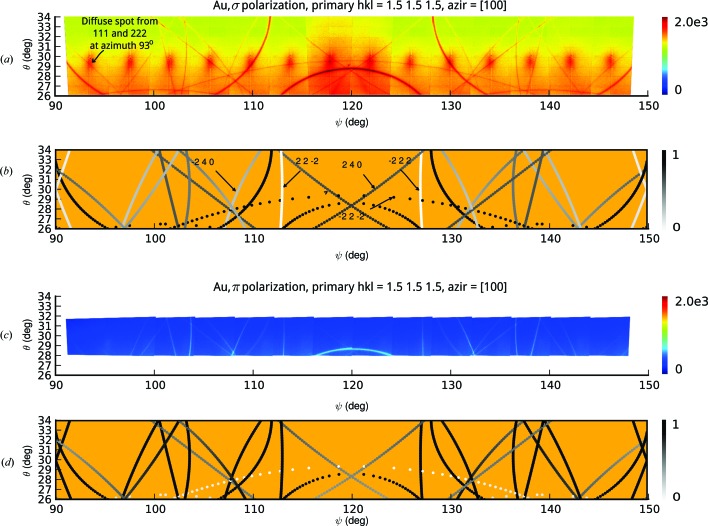
Diffuse multiple scattering in Au(111) at *hkl* = 1.5 1.5 1.5 with an azimuthal reference of [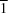
00]. Parts (*a*) and (*c*) show stitched images converted to 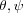; parts (*b*) and (*d*) show the corresponding simulations normalized to the polarization factor.

Figs. 3 ▶(*a*) and 3 ▶(*c*) show experimental data collected for a Au(111) crystal measured at the I16 beamline (Collins *et al.*, 2009 ▶) at Diamond Light Source. The sample was orientated so as to excite the theoretical 1.5 1.5 1.5 Bragg reflection at an energy of 8 keV. Note that the images (*a*) and (*c*) consist of several discrete images taken for different azimuths, which is why the diffuse spots are separated according to the resolution of the stitching or azimuthal step size. The lines, however, flow continuously from image to image for the reasons explained in §2.2. Normally, when the geometry is chosen for a Bragg reflection the beam is incident on the reflecting planes at an angle θ and an azimuth ψ, and this would result in a diffraction spot on the detector. If we assume the beam enters the sample at multiple angles, it becomes convenient to convert the pixels on the area detector (Pilatus 100k in this case) to an array of 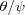 angles. This enables the appropriate stitching for the DMS lines and means that the simulated lines can be conveniently superimposed on the images.

In contrast to Kossel lines, which are isotropic and unpolarized (Gog *et al.*, 1996 ▶), DMS lines exhibit a clear polarization dependence. The two sets of images were taken with σ and π polarization. The 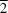
2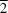 DMS line is clearly visible in the σ geometry and absent in the π geometry; conversely, the 22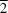 and 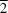
22 DMS lines are absent in the σ geometry and present in the π geometry. Additionally, the 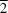
40 is much weaker in the σ geometry. The polarization can be calculated according to
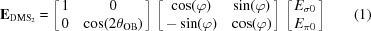
and 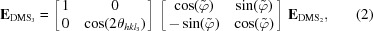
where 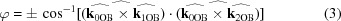
and 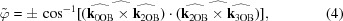
and 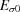 and 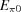 are the elements of the electric field vector perpendicular and parallel, respectively, to the scattering plane of the primary reflection, 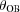 is the off-Bragg angle, corresponding to the beam vectors, 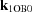 to 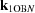, the range of which can be defined by the acceptance angle of the detector.

### 2.4. Line width   

As well as showing the polarization dependence of DMS, Fig. 3 ▶ also exhibits a range of line widths. These can be explained in terms of the sweep of the reciprocal-lattice vectors through the wall of the Ewald sphere. For example, if we assume that the Ewald sphere has a wall thickness due to an energy bandwidth, or that for each reciprocal-lattice vector there is an angular spread of vectors due to the crystal’s mosaicity, or alternatively, there is a spread of vector lengths due to strain in the crystal, then the result will be an intensity distribution as the vectors sweep through the wall of the sphere.

### 2.5. Crystal truncation rod multiple scattering   

**Figure 4:**
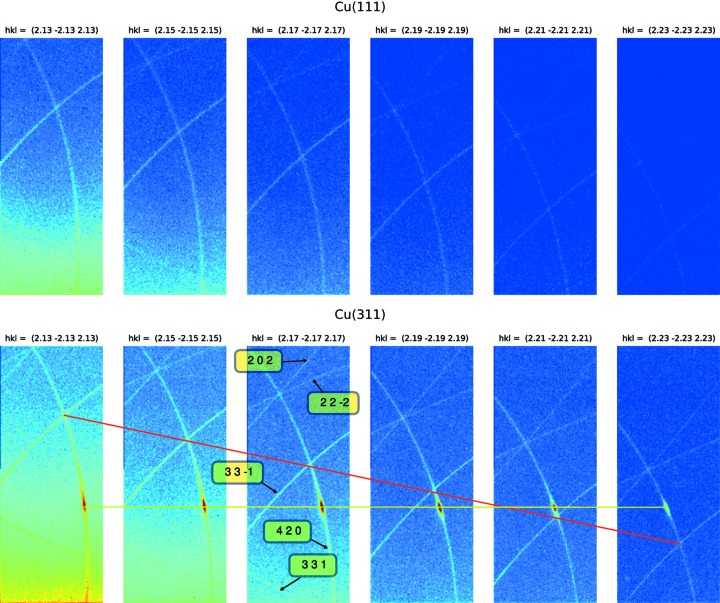
Truncation-rod propagation along the 420 DMS line in Cu(311) over a range of non-integer *hkl* positions at a ψ value of 46° defined with respect to an azimuthal reference of [100]. The images were recorded at an energy of 7.82 keV. The red line is to emphasize the shift in the position of the DMS lines on the detector and the yellow line is to show how the CTR is fixed in the vertical direction but moves in the horizontal direction.

So far we have looked at multiple scattering where the primary reflection is diffuse and is filtered by secondary and tertiary reflections resulting in relatively clean lines. A further component that can make a contribution to the observed signal is the CTR. We measured the DMS lines on two Cu crystals with differing surface cuts, (111) and (311), see Fig. 4 ▶. The top set of images shows the DMS lines for the Cu(111) for a non-integer *hkl* set ranging from 2.13 2.13 2.13 to 2.23 2.23 2.23. The bottom set shows the same measurement for the Cu(311) crystal. The only significant difference between the two crystals is the direction of the surface cut and, therefore, the direction of the truncation rod.

**Figure 5:**
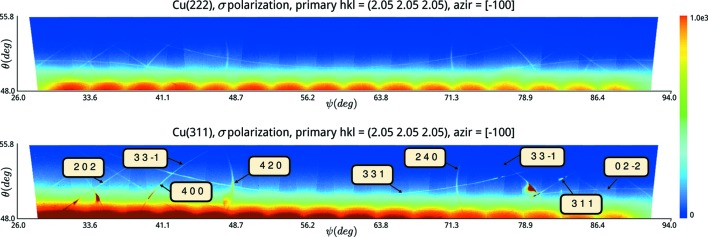
Images showing the influence of surface cut on the DMS signal at 7.82 keV. The top image shows the DMS displayed as a function of ψ about the 2.05 2.05 2.05 primary reflection for the Cu(111) crystal. The bottom image shows the same for the Cu(311) crystal.

Presenting the data for both crystals as a function of azimuth about the 2.05 2.05 2.05 primary reflection reveals the complex interaction between the DMS and the CTR. The CTR and nearby 222 are reinforcing each other and in turn reinforcing the DMS lines (Fig. 5 ▶). While further development is required to model this contribution, the direction of the surface cut has a dramatic effect on multiple scattering and DMS, and should be considered when studying samples in which azimuthal measurements are of interest.

## 3. Applications   

**Figure 6:**
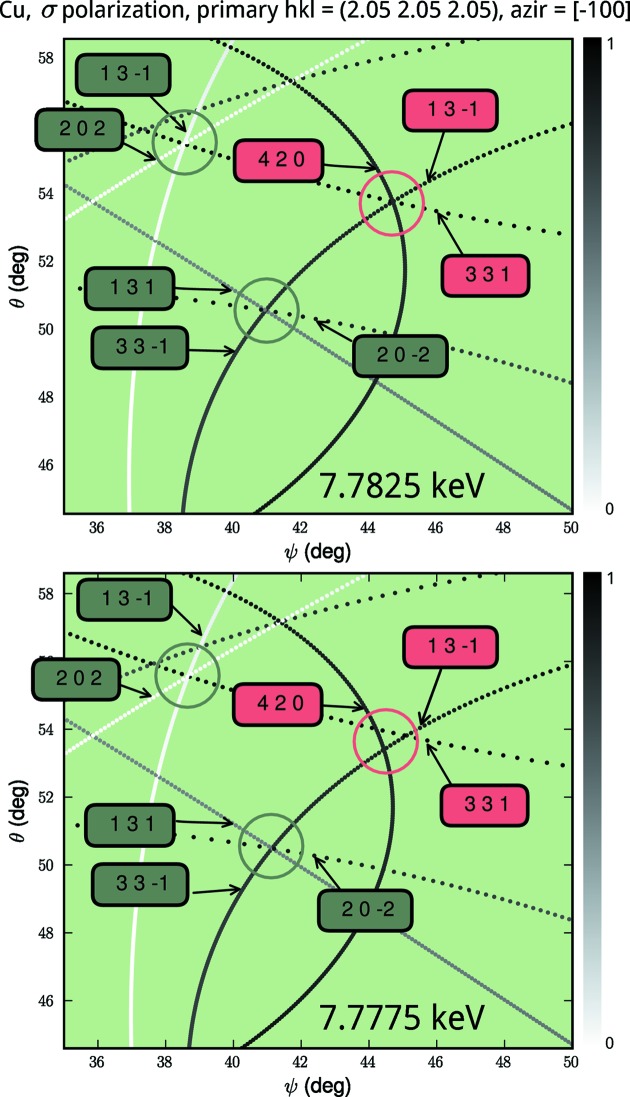
Simulations showing inevitable (green) and accidental (red) triple intersections. An energy of 7.7825 keV was calculated to produce a non-coplanar triple intersection for Cu. A calculation with a shift in energy of 5 eV is also presented to show the persistence of the inevitable intersections and the splitting in the accidental intersections. The grey scale corresponds to the polarization factor.

*Strain measurements*. The divergent-beam method was used by Lonsdale to study diamond, which cannot produce Kossel lines because of the low energy of the C 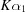 emission line (0.277 keV). Instead, she used the emission lines of Cu, utilizing a highly divergent X-ray tube source. Lonsdale noted the occurrence of geometrically inevitable and so-called ‘accidental’ triple intersections, where the former gives rise to the vanishing determinant 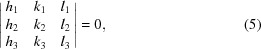
meaning that the reflections are coplanar and the triple intersection occurs at all energies. The accidental intersections, however, occur at precisely known energies and can therefore be used for precise lattice-parameter determination. An example of both cases is shown in Fig. 6 ▶. This method eliminates the geometrical errors in the experimental setup because convergence will occur, regardless of the detector distance or angle.

The precision is limited by the energy bandwidth of the source X-rays, but Lonsdale reported a precision of 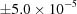
 Å in her measurement of diamond lattice parameters. Until the advent of synchrotron sources, Lonsdale’s intersection method was limited to a few characteristic energies set by the available X-ray tubes. Her method has since been updated by Glazer and his colleagues using synchrotron radiation (Glazer *et al.*, 2004 ▶). Again, however, a target material must be used to provide a source of divergent fluorescence, limiting the scope of the technique to specific source/sample pairings. Furthermore, attaching a foil or secondary material might not always be feasible with certain samples. The method we propose can truly take advantage of the variable energy of synchrotron sources to deliberately choose triple intersections with non-vanishing determinants. Incidentally, coplanar triple intersections can be useful for precise monitoring of structural phase transitions. An obvious advantage over Kikuchi lines, which are tunable, is that measurements can be made in atmosphere and in the presence of electric or magnetic fields.

## 4. Conclusion   

We have demonstrated, for the first time, a new form of diffraction lines. We have highlighted some of the properties of these lines and shown the potential for DMS lines as a strain-measurement technique that truly exploits the tunable capabilities of synchrotron radiation. Furthermore, because we are using X-rays, the measurements can be made in the presence of electric or magnetic fields, thus extending the scope of *in situ* manipulation-type measurements. Finally, because no coating or external divergent source is required, domain mapping could also be achieved using DMS with microfocusing.

## Appendix A DMS code

The code used to calculate the DMS lines along with a description of the mathematical framework is available under the Apache licence and its DOI is 10.5281/zenodo.12866.
